# Dietary diversity and associated factors among pregnant women in the Southern Province of Rwanda: A facility-based cross-sectional study

**DOI:** 10.1371/journal.pone.0297112

**Published:** 2024-02-23

**Authors:** Aline Uwase, Etienne Nsereko, Nirvana Pillay, Jonathan Levin

**Affiliations:** 1 University of Rwanda College of Medicine and Health Sciences, Kigali, Rwanda; 2 University of Witwatersrand School of Public Health, Johannesburg, South Africa; 3 University of Witwatersrand School of Public Health, Pretoria, South Africa; MRC Unit The Gambia at LSHTM, GAMBIA

## Abstract

The inadequate dietary diversity of pregnant women in low- and middle-income countries, including Rwanda, is rising and leading to macro and micronutrient deficiencies. The extent of dietary diversity and the factors contributing to it are unknown in Rwanda. This cross-sectional study, with 612 women who attended antenatal care services in Rwanda’s Southern Province, identified determinants of dietary diversity among pregnant women. A multistage sampling scheme was used in which four districts were sampled, thereafter one urban and one rural health centre was sampled in each district and finally, a systematic sample of pregnant women was selected in each sampled health centre. Dietary diversity was measured using Minimum Dietary Diversity for Women (MDD-W), and multiple logistic regression models were fitted to identify factors associated with dietary diversity. Only 44.1% (95% confidence interval (CI) of [40.1%, 48.0%]) of participants had adequate dietary diversity. Approximately 95.4% of participants consumed grains, white roots, and tubers. The food groups that were the least consumed consisted of eggs (n = 99, 16.4%), as well as those consisting of milk and milk products (n = 112, 18.5%). The factors which were positively associated with dietary diversity were owning a radio (adjusted odds ratio [aOR] = 1.90 [95% CI 1.27, 2.85]), maternal education (aOR = 1.85 [95% CI 1.28, 2.65]), having a kitchen garden (aOR = 1.69 [95% CI 1.11, 2.57]) and nutrition knowledge score (aOR = 1.45 [95% CI 1.21, 1.74]) for a five-point increase in nutrition knowledge score. The factors negatively associated with dietary diversity include food insecurity, which reduced the odds of dietary diversity (aOR = 0.19 [0.07, 0.50]) per five-unit increase in food insecurity. Furthermore, the odds of adequate dietary diversity were lower among urban residents than rural residents (aOR = 0.69 [0.47, 1.03]). The household size was associated with dietary diversity with the odds of dietary diversity decreasing by 12% for a five-unit increase in household size (aOR = 0.88 [0.79; 0.99]). 23% had poor nutritional status, indicated by their mid-upper arm circumference (MUAC; < 23 cm). Enhanced nutritional education is needed to improve the nutritional knowledge of this population with particular emphasis on the consumption of animal-source foods. Sensitisation activities promoting ownership of kitchen gardens and radios could improve dietary diversity among Rwanda’s pregnant women.

## Introduction

Pregnancy is a significant and nutritionally demanding period of a woman’s life [1] because the physiological changes that ensure foetal growth and development lead to higher nutritional demands during pregnancy. The World Health Organization (WHO) recommends that pregnant women adopt healthy dietary practices by consuming adequate protein, carbohydrates, vitamins, and minerals [2]. To comply with this recommendation, the National Food and Nutrition Policy of Rwanda recommends intervention packages that combine nutritional education about consuming nutritious and diverse foods and improved agricultural practices. These agricultural practices include 1) kitchen gardens to promote access to minerals and vitamins through vegetables; and 2) livestock ownership, mainly through the Girinka program (meaning “one cow-per-poor family”) to promote access to proteins and other nutrients [3].

These National Food Nutrition Policy interventions were introduced because adherence to a diverse daily diet is the main strategy for complying with the WHO recommendation [4]. Dietary diversity is defined as the ‘number of food types consumed through and within food groups during a specified period’ [5]. It is frequently reported as a proxy indicator of diet quality and can be summarised using the number of food groups consumed during the previous 24 hours [6]. Dietary diversity promotes good health by preventing micronutrient deficiencies [7]. Inadequate dietary diversity, which is consuming fewer than five food groups in the previous 24 hours, is prevalent among pregnant women in low- and middle-income countries [8].

Studies report a high prevalence of poor dietary diversity during pregnancy in Oromia, Ethiopia (74.6%) [7], North East Ethiopia (68.6%) [9], and Tanzania (54%) [10]. A study in Nigeria found that many women consumed vegetables, and among them, half practised food restrictions with regard to meat, eggs, and fish due to food taboos [11]. Women in low- and middle-income countries have a starch-based diet that is largely deficient in animal-source foods and vegetables and fruit, which are rich in protein and minerals needed for the foetus [12].

When pregnant women do not diversify their diets, it leads to nutritional deficits, which is a public health concern in low- and middle-income countries. Maternal undernutrition varies between 10% and 19% in many countries of sub‑Saharan Africa, as well as in south‑eastern and south‑central Asia [13]. In developing countries, more than half of all pregnant women have nutritional anaemia [14]: The prevalence of anaemia among reproductive women is 52.5% in the WHO South Asian region and 46% in the African region [14].

Poor nutritional status during pregnancy negatively affects the physiological growth of the foetus and increases the risk of preterm delivery, low birth weight, and maternal morbidity and mortality [15]. The effect of deficient nutritional intake during pregnancy significantly shapes foetal health and exposure to some adulthood non-communicable diseases. However, high dietary diversity results in sufficient nutrients to ensure proper foetal growth [16].

Different factors contribute to dietary diversity during pregnancy. Studies report that factors associated with good dietary diversity during pregnancy include adequate knowledge of proper nutrition [17], middle to high income, and demographic characteristics, such as maternal age, education, occupation, and marital status [18]. In addition, food security, including both accessibility and affordability, is associated with dietary diversity [19]. Ownership of a radio, livestock, and a home garden positively affects dietary diversity [20]. Furthermore, cultural beliefs and food taboos can negatively affect the dietary diversity of pregnant women because these taboos apply to green vegetables, cheese, yoghurt, green peppers, sugar cane, and other nutritious foods [21, 22].

There is a shortage of studies on the factors associated with dietary diversity among pregnant women in Rwanda. A 2017 study conducted in Kigali City reported that 50% of study participants did not achieve the Minimum Dietary Diversity for Women (MDD-W) score [23]; however, the study did not assess the factors contributing to this low score. Addressing this gap is critical because the 2019–2020 Demographic and Health Survey of Rwanda report that anaemia among pregnant women has increased from 17% in 2010 to 25% in 2020, and the Southern Province accounted for the highest prevalence of anaemia [24]. Approximately 9% of the women in the Southern Province were underweight with a body mass index (BMI) < 18.5, compared to 6% of women nationally [25]. Poor maternal dietary diversity could have contributed to this high prevalence of anaemia and low BMI among pregnant women. Therefore, this study assessed the status of dietary diversity, its determinants, and the nutritional status of pregnant women in the Southern Province of Rwanda.

## Methods and materials

### Study design and setting

This cross-sectional study was conducted with pregnant women receiving antenatal care (ANC) services. It was conducted in the Southern Province of Rwanda, which shares its borders with Kigali City and the Northern Province to the North, the Republic of Burundi to the South, the Eastern Province to the East, and the Western Province to the West. In 2017, the Southern Province was ranked the second among five Provinces in having household food insecurity (20.5%) and the third in the poverty rate, which increased from 12.9 to 16.9% [26]. According to the 2014–2015 Demographic and Health Survey of Rwanda and the 2019–2020 Demographic and Health Survey results, the Southern Province accounted for the highest prevalence of underweight and anaemic pregnant women. Data were collected within two months, from May 16^th^ to July 18^th^ 2022.

### Study population, sample size determination and sampling procedure

Adult pregnant women (> 18 years) who self-reported being healthy in their second or third trimester were recruited to participate in the study. Women who were not healthy were excluded because some chronic diseases require special diets, which could affect dietary diversity. Pregnant women in their first trimester were excluded because most pregnant women are physiologically unstable and experience dietary disturbances, which could affect their dietary diversity.

A sample size of 612 pregnant women was chosen as the appropriate sample size to estimate the proportion of pregnant women with adequate dietary diversity to within 5%, using the formula [27]: $n=\frac{Z^{2}P(1-P)}{d^{2}}$

We assumed that the proportion of women with adequate dietary diversity would be approximately 50%, which would make our sample size calculation slightly conservative. A sample size of 385 was considered sufficient to estimate the proportion to within ± 5% (as measured by the 95% CI for the proportion). This sample size assumed that a simple random sample of women was recruited; thus, allowances needed to be made for any clustering effects of women within a clinic. Assuming a moderate design effect of 1.5 increased the sample size to 578, and allowing for a 6% of refusal rate led to the final sample size of 612.

A multistage sampling scheme was used to sample 612 pregnant women. At the first stage four out of the eight districts in the Southern Province were selected using simple random sampling. At the second stage in each of the four sampled districts one urban health centre and one rural health centre were selected using stratified random sampling (with the strata being “rural” and “urban”). This gave a selected sample of eight health centres. Since each health centre was expected to receive about 200 pregnant women monthly, this would give an expected total of approximately 1600 pregnant women. Since we required a sample of 612 women, the sampling interval was approximately 3. In each of the sampled health centres a systematic sample of pregnant women was selected with a random start after which every third woman was sampled.

### Data collection process

Before data collection, eight interviewers (nurses and midwives) were trained for three days to collect quantitative data including anthropometric measurements. They were informed about the study’s purpose, data collection process and data collection tools. Data collection tools were translated into the local language (Kinyarwanda), and they were pre-tested in health centres which were not part of the study to ensure that all questions were clear and interviewers had the same understanding of them. After the pre-test, a few questions were adjusted for clarity. In addition, data collection tools were anonymous and Codes were used to identify the participants. Two interviewers were allocated to each district to collect data from the health centres located in that particular district. Interviewers approached the healthcare providers in the ANC service and requested the list of pregnant women registered for ANC consultations. They approached every third pregnant woman and briefly explained the study. Thereafter, the nutritional status of each pregnant woman was evaluated by measuring their MUAC using non-stretchable MUAC tape designed for adults. The literature reports that MUAC is the easiest anthropometric measurement of nutritional status in pregnant women, since it is easy to measure, does not require much training or resources and unlike BMI does not require calculations. Most importantly, MUAC is a better nutritional status indicator than BMI because it changes minimally during pregnancy [28]. Two researchers independently assessed the MUAC value of the non-dominant arm to the nearest 0.1 cm without clothing on the arm [29]. The MUAC of each pregnant woman was recorded, and in case of a difference in measurements between two researchers, the average MUAC was considered. The cut-off MUAC of 23 cm was used as it has been recommended by different authors, especially for Asian and African pregnant women [30]. Pregnant women were assigned to one of two categories: poor nutritional status (MUAC < 23 cm) or good nutritional status (MUAC ≥ 23 cm) [30].

## Measurement of study variables

### Outcome variable

Dietary diversity was assessed using the MDD-W adapted from the Food and Agriculture Organisation guidelines [31] by creating a list of foods that are locally available. The MDD-W indicator consists of 10 food groups; participants receive a score of 1 for a given food group if she consumes at least one item in that food group; otherwise, the participant receives a score of zero for that food group. The assessment was conducted by asking participants to report all drinks and foods they had consumed the previous day (the last 24 hours), and a probing technique was used to help pregnant women recall all foods and drinks consumed. A list of 56 local foods was included in the questionnaire and sorted into 10 food groups. This list is provided as a supporting file in S1 Table. The total dietary diversity score was calculated and dichotomised as adequate dietary diversity (consumption of five or more food groups) or inadequate dietary diversity (consumption of less than five food groups) [31].

### Explanatory variables

Data collected on sociodemographic characteristics included occupation, age, marital status, education, and religion, and data on obstetric characteristics included gestational weeks, parity, and the number of ANC visits. The socioeconomic section of the questionnaire assessed participants’ ownership of a radio, a mobile phone, and livestock, and their ‘ubudehe’ category, which is a social stratification based on household income, ranging from the highest income of the richest households, to the lowest income of the poorest and most vulnerable households in society.

Nutritional knowledge was assessed using the questionnaire adapted from the Food and Agriculture Organisation’s Knowledge, Attitude, and Practice Manual [32]. It included 13 items, which assessed knowledge of adequate nutrition, recognition and prevention of anaemia during pregnancy, the importance of nutrition during pregnancy, and weight gain, resulting in a score between 0 and 32. Nutritional knowledge was classified as poor (< 50%), moderate (50%-74%), and good (≥ 75%). The items assessed are provided in S2 Table.

We measured the household’s food security using the Household Food Insecurity Access Scale designed by Food and Nutrition Technical Assistance [33]. The scale consists of nine ‘occurrence questions’ and nine questions related to the frequency of occurrence. The occurrence questions characterise different dimensions of food insecurity, and the frequency of occurrence question is skipped if the participant reports that the corresponding occurrence has not occurred during the previous four weeks (30 days). For descriptive statistics, levels of food insecurity included in the Household Food Insecurity Access Scale ranged from no insecurity (score < 2), mild insecurity (score 2–10), moderate insecurity (score 11–17), and severe insecurity (score > 17). The actual score was used for the multivariable analysis. Questions pertaining to the policy were adapted from the existing Rwandan program to assess the availability of kitchen gardens and being part of the Girinka program (i.e. the one cow per low-income family program).

Social support was assessed using the Maternity Social Support Scale designed by Webster et al. [34]. It covers six items and assesses family support, such as the help from husband, friendship network, conflict with husband/male partner, feeling controlled by husband/male partner, and feeling loved by husband/male partner. Every item was assessed on a five-point Likert scale ranging from 1 to 5 points with a possible maximum score of 30. For descriptive purposes, Maternity Social Support Scale was categorised as low social support (0–18), moderate social support (19–24) and high social support (25–30). However, the actual score was used in data logistic regression.

### Data processing and analyses

Data were cleaned using STATA Release 16, which was also used to analyse the data by computing descriptive statistics, namely frequencies and percentages for the categorical variables and means and standard deviations (SD) for quantitative variables. We did a complete case analysis; those who had fasted on the previous day were excluded from the analysis. In the whole study, there were very few missing values. The procedure explained below was used to select the final multiple logistic regression model for dietary diversity. Initially univariable logistic regression models were fitted to identify factors that were potentially associated with dietary diversity, using a liberal screening p-value of 0.20 to ensure that all potential confounders were investigated. In addition, variables that were part of the design (district and rural/urban) and variables previously identified in the literature as being associated with dietary diversity (age and wealth category) were included in the final model irrespective of statistical significance. The quantitative explanatory variables were included in the model as continuous variates, with fractional polynomials used to test for any non-linearity in their effect, as several authors have pointed out the disadvantage of categorising continuous exposure variables [35–38]. Once the pool of potential explanatory variables had been identified, backward elimination with a nominal p-value for exclusion of 0.05 was used to select the final model, with district, rural/urban, age and wealth category fixed in the final model, While backward elimination and other stepwise procedures have a number of well-known disadvantages, it is recommended for analyses such as this in which there is no primary exposure of interest, and it has the relative advantage that negatively confounded sets of variables are less likely to be omitted from the model since all important explanatory variables are included in the initial model. This recommendation can be found in chapter 10 of Regression Methods in Biostatistics: Linear, Logistic, Survival and Repeated Measures Models. Second Edition by Vittinghoff E and colleagues in (2012). Due to the small number of clusters (8 health centres) in our model, we fitted terms for district and rural/urban rather than adjusting for clusters as random effects. The impact of factors on dietary diversity was expressed as odds ratios with 95% confidence limits. Interactions involving the main exposures of interest (ownership of a radio, having a kitchen garden, food insecurity and nutritional knowledge) were investigated but none were found to be statistically significant. Potential multicollinearity was investigated through looking at the variance inflation factors for the final model, but no multicollinearity was found. In addition, we checked for goodness of fit of the final model using the Hosmer-Lemeshow goodness of fit test, which showed no evidence of lack of fit. We computed the Cronbach alpha for food insecurity which became 0.93 and maternal social support, which became 0.74 indicating acceptable reliability.

### Ethics approval and consent to participate

The implementation of this study complied with the Declaration of Helsinki. This study was a part of a research project that has been approved by relevant authorities. Ethical clearances were secured from the Institutional Review Board of the University of Rwanda, where the study was conducted (287/CMHS IRB/2021), and the Human Research Ethics Committee (Medical) of the University of Witwatersrand, where the corresponding author is registered (M211046). Before the survey, participants provided written consent to be part of the study after receiving comprehensive details about the purpose, benefits, and risks of participation. Participants were also assured that their participation was voluntary and that the information they provided would remain confidential. They signed the consent form or provided fingerprints (for illiterate) as they were above18 years old.

## Results

### Sociodemographic, economic, and obstetric characteristics of participants

Of the 612 pregnant women who participated in this study, six who reported that they had fasted 24 hrs before the interview date were excluded from the analysis because, in this case, the previous 24 hours were deemed not representative of their usual dietary diversity practices. Therefore, the data analysis included 606 participants. The mean age of the participants was (28 ± 6.5 SD) years. A large proportion of participants *(*n = 267) was between 25 and 34 years old, and 138 (51.7%) of them presented with inadequate dietary diversity. A total of 402 participants attended primary school; among them, 254 (63.2%) had inadequate dietary diversity. A total of 498 participants were married and more than half of them had inadequate dietary diversity (n = 265, 53.2%). Many participants (n = 243) reported being in the second wealth category (poor), and among them, 140 (57.6%) had inadequate dietary diversity; 306 women were classified as experiencing severe food insecurity, and the majority of these women had inadequate dietary diversity (n = 203, 66.3%). As for their clinical characteristics, most of the participants attended two to three ANC visits (n = 394) and 212 (53.8%) had inadequate dietary diversity (Table 1).

**Table 1:** Sociodemographic, economic, and obstetric characteristics of participants.

		Dietary Diversity		
Factor	Category	Adequate n (%)	Inadequate n (%)	Total (N = 606)
**Overall**		267(44.1)	339(55.9)	
**Age (years)**	**Mean (SD)**	29(6.7)	28(6.2)	28(6.5)
	18–24	91 (41.2)	130 (58.8)	221
	25–34	129(48.3)	138 (51.7)	267
	≥ 35	47(39.8)	71(60.2)	118
**Education**	None	12(35.3)	22(64.7)	34
	Primary	148(36.8)	254(63.2)	402
	Secondary and above	107(62.9)	63(37.1)	170
**Religion**	Christian	251(43.4)	327(56.6)	578
	Other	16(57.1)	12(48.9)	28
**Occupation**	Housewife	204(41.5)	287(58.5)	491
	Paying job	63(54.8)	52(45.2)	115
**Marital status**	Married	233(46.8)	265(53.2)	498
	Single	34(31.5)	74 (68.5)	108
**Household size**	1–3	136(47.1)	153(52.9)	289
	4–6	105(42.3)	143(57.7)	248
	≥ 7	26(37.7)	43(62.3)	69
**District**	Gisagara	55(36.2)	97(63.8)	152
	Huye	57(38.3)	92(61.7)	149
	Kamonyi	103(67.3)	50(32.7)	153
	Ruhango	52(34.2)	100(65.8)	152
**Residential area**	Rural	142(47.0)	160(53.0)	302
	Urban	125(41.1)	179(58.9)	304
**Gestational weeks**	13–27	100(45.25)	121(54.75)	221
	28–44	167(43.4)	218(56.6)	385
**Parity**	0–1	155(47.8)	169(51.2)	324
	2–3	81(40.1)	121(59.9)	202
	> = 4	31(38.7)	49(61.3)	80
**ANC visits**	0–1	59(37.3)	99(62.7)	158
	2–3	182(46.2)	212(53.8)	394
	> = 4	26(48.2)	28(51.8)	54
**Ubudehe category**	Poorer	55(42.9)	73(57.1)	128
	Poor	103(42.4)	140(57.6)	243
	Richer	101(45.3)	122(54.7)	223
	Unknown	8 (66.67)	4 (33.3)	12
**Radio**	No	93(32.1)	197(67.9)	290
	Yes	174(55.1)	142(44.9)	316
**Telephone**	No	34(26.9)	92(73.1)	126
	Yes	233(48.5)	247(51.5)	480
**Access to agricultural land** (n = 605)	No	114(47.9)	124(52.1)	238
	Yes	153(41.7)	214(58.3)	367
**Kitchen garden** (n = 605**)**	No	74(34.9)	138(65.1)	212
	Yes	193(49.1)	200(50.9)	393
**Food security**	Food security	61 (73.5)	22(26.5)	83
	Mild food insecurity	54(58.7)	38(41.3)	92
	Moderate food insecurity	49(39.2)	76 (60.8)	125
	Severe food insecurity	103(33.7)	203(66.3)	306
**Nutritional knowledge**	Poor	54(31.1)	120(68.9)	174
	Moderate	94(45.6)	112(54.4)	206
	Good	119(52.6)	107(47.4)	226
**Social support**	Low	36(27.7)	94(72)	130
	Moderate	123(49.6)	125(50.4)	248
	High	108(47.4)	120(52.6)	228

### Dietary diversity of pregnant women

This study found that the mean dietary diversity score was 4.40 (SD = 2.14). Details about dietary diversity score are provided in S3 Table. Less than half of the participants had adequate dietary diversity (n = 267, 44.1%), with a 95% CI of [40.1%, 48.0%]. The most consumed food group was that consisting of grains, white roots, tubers (n = 584, 96.4%), and pulses (n = 475, 78.4%). The food groups that were the least consumed consisted of eggs (n = 99, 16.4%), as well as those consisting of milk and milk products (n = 112, 18.5%). More details are provided in (Table 2).

**Table 2:** Dietary diversity of pregnant women.

Food groups	Frequency (n = 606)	%
Grains, white roots and tubers, and plantains	584	96.4
Pulses (beans, peas and lentils)	475	78.4
Nuts and seeds	186	30.7
Dairy	112	18.5
Meat, poultry and fish	251	41.4
Eggs	99	16.3
Dark green leafy vegetables	365	60.2
Other vitamin A-rich fruits and vegetables	213	31.5
Other vegetables	201	33.2
Other fruits	207	34.12
**Dietary diversity adequacy**		
Adequate > = 5	267	44.1
Inadequate <5	339	55.9
**Mean dietary diversity score**	**SD**	
4.40	2.14	

SD: Standard Deviation

### Nutritional status and dietary diversity of pregnant women in the Southern Province

In this study, 141 (23.1%) pregnant women had a MUAC < 23 cm, indicating poor nutritional status. The mean MUAC was 24.8 cm (SD = 2.64) and the proportion of participants with poor nutritional status was higher among those with inadequate dietary diversity (25.7% vs 19.1%), which was a marginally significant (*p* = 0.053) difference (Table 3).

**Table 3:** Nutritional status and dietary diversity.

Variable	Category	Nutritional status		Chi-square	*p*-value
		Poor (MUAC < 23 cm) n (%)	Good (MUAC ≥ 23 cm) n (%)		
Dietary diversity	Inadequate	87 (25.7%)	251 (74.3%)	3.7336	0.053
	Adequate	51 (19.1%)	216 (80.9%)		

MUAC, mid-upper arm circumference

### Determinants of dietary diversity of pregnant women in the Southern Province of Rwanda

The results of fitting multiple logistic regression models to find factors associated with adequate dietary diversity are summarised in Table 4. The adjusted odds ratio (aOR) for owning a radio was 1.90 with a 95% CI of [1.27, 2.85] and having a kitchen garden (aOR = 1.69 [1.11, 2.57]) increased the likelihood of adequate dietary diversity. Food insecurity was associated with inadequate dietary diversity, as indicated by the aOR < 1 for adequate dietary diversity (aOR = 0.19 [0.07, 0.50]). Other factors associated with dietary diversity were maternal education (aOR = 1.85 [1.28, 2.65]) nutritional knowledge (aOR = 1.45 [1.21, 1.74]), household size (aOR = 0.88 [0.79;0.99]), and urban residence (aOR = 0.69 [0.47, 1.03]) showing a significant difference in dietary diversity among the four districts (*p* < 0.001) with the odds of dietary diversity being highest in Kamonyi district (Table 4). The overall variance inflation factor was 1.43 and the highest was 2.65; the Hosmer-Lemeshow goodness of fit test showed no evidence of a lack of fit (P = 0.469).

**Table 4:** Determinants of dietary diversity of pregnant women in the Southern Province of Rwanda.

Factor (n = 612)	Category	Unadjusted OR [95% CI]	*p*-value	Adjusted OR [95% CI]	*p*-value
**District**	Gisagara	1		1	<0.001
	Huye	1.09[0.68,1.74]	0.710	1.43[0.81, 2.48]	
	Kamonyi	3.63[2.26, 5.82]	<0.00	4.29[2.43, 7.56]	
	Ruhango	0.91[0.57, 1.46]	0.719	1.33[0.73, 2.42]	
**Residence**	Rural	1		1	0.005
	Urban	0.79[0.57, 1.09]	0.144	0.59[0.40, 0.87]	
**Age**	Per one-year increase	.99[0.97, 1.02]	0.526	1.01[0.98;1.04]	
**Ubudehe category**		0.97[0.79, 1.18]	0.778	0.93[0.73;1.18]	
**Education-cubed**		1.03[1.01, 1.04]	<0.001	1.02[1.01, 1.03]	0.003
**Religion**	Christian	1			
	Others	1.76[0.82, 3.80]	0.004		
**Occupation**	Housewife	1			
	Paying Job	1.66[1.10, 2.49]	0.014		
**Marital status**	Married	1			
	Single	0.52[0.33, 0.81]	0.145		
**Household Size**	Per one member increase	0.82[0.64, 1.04]	0.115	0.88[0.79;0.99]	0.03
**Gestational week**	Per one unit increase	0.99[0.97, 1.02]	0.811		
**Parity**	Per one child increase	0.80[0.63, 1.00]	0.058		
**ANC visits**	Per one visit increase	1.30[0.98, 1.74]	0.065		
**Radio**	No	1		1	< 0.001
	Yes	2.64[0.89, 3.67]	<0.001	1.85[1.25, 2.75]	
**Telephone**	No	1			
	Yes	2.60[1.69, 4.00]	<0.001		
**Access to agricultural land**	No				
	Yes	0.78[0.56, 1.08]	0.147		
**Kitchen garden(n = 611)**	No	1		1	0.009
	Yes	1.81[1.28, 2.55]	0.001	1.83[1.21, 2.76]	
**Food insecurity**	Per 5 units increase	.065[0.03, 0.14]	<0.001	0.16[0.06, 0.40]	<0.001
**Nutritional knowledge** **Social support**	Per 5 units’ increase Per 5 units increase	1.41[1.22, 1.62] 5.7[1.91; 16.81]	<0.001 0.002	1.51[1.27, 1.81]	<0.001

OR: Odd ratio

## Discussion

This study assessed the dietary diversity of pregnant women who attended ANC services in the Southern Province of Rwanda and investigated the factors associated with adequate dietary diversity. Less than half of all the pregnant women (44.1%) met the minimum dietary diversity criterion of consuming at least five food groups in the previous 24 hours. The determinants of dietary diversity among the study participants include owning a radio, having a kitchen garden, food insecurity, nutritional knowledge, maternal education, household size, and being urban residents.

The proportion of pregnant women who achieved adequate dietary diversity (44.1%) was lower than the proportions reported in studies conducted in Hosanna Town, Ethiopia (57.4%) [20], Southwest Ethiopia (48.3%) [39], Nepal (55%) [40], and the Gurage Zone, Ethiopia (57.9%) [18]. However, the proportion was higher than those reported in studies conducted in North East Ethiopia (31.4%) [9] and Oromia Ethiopia (23.6%) [7]. This discrepancy can be explained by differences in study periods and the geographical and sociocultural differences between the countries.

Grain, white root, and tuber foods were more consumed (96.4%) than other food groups. This finding is consistent with the literature that shows that the diet of pregnant women in low- and middle-income countries is mainly cereal-based and deficient in animal-source foods [41] It is also similar to the findings of a study conducted in Southwest Ethiopia that report that 99.8% of pregnant mothers consumed food consisting of white roots, grains, and tubers [42]. Food groups that were less consumed by the participants in this study include eggs (16.3%) and milk and milk products (18.5%), which may be related to the higher price of animal-source foods. Thus, some pregnant women may have limited their consumption of animal products to holidays (Christmas and Easter) and ceremonies. However, these days were excluded from the data collection. Furthermore, some cultures restrict animal-source foods during pregnancy because these foods are considered taboo during pregnancy; for example, some pregnant women in Tanzania and South Africa restrict meat, milk, and eggs due to cultural norms [43, 44]. Similarly, a high consumption of cereal-based foods was reported in a study conducted in Tanzania [10]. However, this finding was different from the results of a study conducted in East Gojjam, Ethiopia, that report that legumes and seeds were the most frequently consumed food groups (85.5%) [45]. This difference may be due to self-reporting differences, geographical location, and seasonal variation. This finding implies that pregnant women in the Southern Province of Rwanda are likely to have nutritional deficiencies and poor pregnancy outcomes because they do not consume a sufficient amount of animal-source foods containing higher quality protein and vital micronutrients essential for positive pregnancy outcomes and the growth and development of infants [46].

Maternal education was positively associated with optimal dietary diversity in this study, which may be related to participants’ greater access to different sources of nutritional information that improved their nutritional knowledge, and hence, their dietary diversity. Education is a proxy measure of socioeconomic status, which could influence the ability to afford various types of food, and less education and a lower socioeconomic status could interfere with dietary diversity because of a lack of basic information on nutrition, regardless of other circumstances. This finding is similar to the results of a study conducted in Brazil that report that socioeconomic factors, including a high educational level, were associated with varied dietary patterns [47]. According to this study conducted in Brazil, a varied dietary pattern consisted of various food items, such as ‘grains, cereals and tubers’, ‘bread, cakes and cookies’, and fruits and vegetables. Similarly, a study conducted in Ethiopia among pregnant women found that secondary and tertiary education of mothers were associated with adequate dietary diversity [7].

Household size negatively affects the dietary diversity of pregnant women. Increased household size decreased the odds of adequate dietary diversity among pregnant women. It may be due to the fact that a large household decreases the quantity and quality of food consumed per person because the food is shared among many people. Consequently, many families prioritise food quantity over quality, leading to poor diversification of consumed food. It is consistent with other studies conducted in South Africa and Nigeria [48, 49].

Owning a radio was positively associated with the dietary diversity of pregnant women in this study. Pregnant women who owned a radio were two times more likely to vary their diet than those who did not own a radio. This finding may be because owning radio could expose pregnant women to nutritional information that can affect their dietary practices. The economic status of participants who owned a radio may have been higher than that of those who did not have a radio, which could have increased their access to information, and thereby, improved their dietary diversity. However, studies with appropriate design could help identify such association. This finding is similar to the results of a secondary analysis of data from three sub-Saharan African countries (Gambia, Liberia, and Rwanda) that report that mothers who had access to radios were more likely to diversify the diets of their children [50].

Having a kitchen garden was positively associated with dietary diversity. The odds of pregnant women who owned kitchen gardens having good dietary diversity were 1.59 times higher than for pregnant women who did not own a kitchen garden. This finding may be explained by the greater likelihood of a kitchen garden owner consuming different types of vegetables without purchasing them, and using the money that might have been used to buy vegetables to purchase other food groups. Furthermore, kitchen garden owners can sell their excess vegetables and use their earnings to buy different varieties of food groups. A randomised controlled trial conducted in Tanzania to evaluate the effect of home gardens on dietary diversity among women observed that the women in the intervention group consumed more food groups than those in the comparison group [51]. The consistency of these results between the two studies can be attributed to the similarity between the study participants and their geographical locations.

In the present study, it was found that food insecurity predicts adequate dietary diversity. This means a negative association where a decrease in food security was associated with a decrease in the odds of adequate dietary diversity, which was most likely because food insecurity decreases household food availability and accessibility, resulting in poor dietary diversity practices. Limited food availability affects intra-household food allocation because pregnant women often prioritise the nutrition of their family members over their own nutritional requirements. Studies conducted in Malawi and Ethiopia similarly found that women from households with moderate and severe levels of food insecurity had a low dietary diversity score and inadequate dietary diversity compared to women from households with low levels of food insecurity [33, 52, 53].

This study highlighted the positive relationship between nutritional knowledge and dietary diversity. Having nutritional knowledge increased participants’ likelihood of exhibiting good dietary diversity. These results are consistent with those of studies conducted in Malawi [54], Bangladesh [55], and India [56], which found that having adequate knowledge of optimal nutrition during pregnancy translates to the practice of consuming food from different food groups. Interestingly, the urban residence was negatively associated with adequate dietary diversity. Pregnant women treated in urban health centres were less likely than those from rural health centres to achieve adequate dietary diversity. These results may be explained by the many opportunities for women in rural areas who are mainly farmers raising livestock to access a variety animal- and non-animal-source foods. On the other hand, pregnant women from urban health centres may have to purchase all of the food they consume due to limited space for farming, making it more difficult to access various food groups. These findings suggest that pregnant women in urban health centres with a low socioeconomic status require more attention and encouragement from staff to help them to improve their dietary diversity, which may be poor because of the high cost of living in urban areas. Although this finding is consistent with that of a study conducted in Nigeria [49], it differs from a study conducted in Ethiopia, in which dietary diversity was found to be better among residents of an urban area [57]. These cross-country differences may reflect the individual participants’ characteristics and differences related to geographical locations.

### Policy and practice implications

The findings of this study supports the recommendations of the NFNP by calling for interventions to improve the dietary diversity of pregnant women in Rwanda. Different stakeholders should be involved in the implementation of nutrition-sensitive and nutrition-specific interventions. Agricultural interventions designed to foster kitchen garden ownership could help improve the dietary diversity of pregnant women, and nurses and midwives could enhance the nutrition education offered during ANC visits by emphasising the diversification of diets and the importance of consuming animal-source foods during pregnancy. Specific nutritional messages should be disseminated through different media, including radio.

### Limitations

This study was based in health facilities; therefore, the findings are not generalisable to the entire population. Social desirability and recall bias could have occurred because we requested pregnant women to recall information and provide self-reports of what they consumed within the previous 24 hours. However, we used probing to mitigate this potential bias. In addition, the food consumed during the preceding 24 hours might not have been representative of the participants’ routine diet. We excluded weekends and days with ceremonial major events to minimise such differences. The nutritional status of the participants was measured using the MUAC due to resource constraints; however, it would have been better to combine the MUAC results with the results of other biological indicators, such as haemoglobin level. The findings of this study should be triangulated with the qualitative study to explore whether there is a cultural influence on the dietary diversity of pregnant women in Rwanda.

## Conclusion

More than half the participants in this study had inadequate dietary diversity. Many pregnant women consumed grains, white roots, and tubers, whereas few pregnant women consumed animal-source foods. Factors such as maternal education, food insecurity, ownership of a radio or kitchen garden, and having good nutritional knowledge were determinants of dietary diversity. Nutrition education is needed to emphasise the importance of animal-source foods and affordable alternatives to access them. Home visits for community sensitisation to raise awareness of the importance of owning a kitchen garden and radio could help improve the dietary diversity of pregnant women. Furthermore, research studies measuring the micronutrient levels of pregnant women in Rwanda’s Southern Province would be very important. There should be studies conducted to validate the MDD-W/Household Food Insecurity Access Scale tools in the Rwandan context.

## Supporting information

S1 Table Description of local foods included in the dietary diversity questionnaire. (PDF)

S2 Table Description of questions used to assess the nutritional knowledge. (PDF)

S3 Table Detailed dietary diversity scores. (PDF)
