# Supplementary material for: Dietary diversity and associated factors among pregnant women in the Southern Province of Rwanda: A facility-based cross-sectional study
Source: PLoS One. 2024 Feb 23;19(2):e0297112. doi: 10.1371/journal.pone.0297112 (PMC10889653; doi:10.1371/journal.pone.0297112)
Supplement: S1 Table — (PDF) [file pone.0297112.s001.pdf]

List of local foods and their food groups

Food group	Food item
1. Grains, white roots and tubers, and plantains	corn/maize
	Sorghum
	Rice, wheat
	breads, sorghum and maize paps, porridge
	Cassava, White sweet potato , Irish potatoes, yams, taro
	Unripe banana
2. Pulses (beans, peas and lentils)	Beans, Peas and soya
3. Nuts and seeds	Ground nuts, sunflower, sesame, and pumpkin seeds
4. Dairy	Milk, soft or hard chees
5. Meat, poultry and fish	Fresh/dried small or big Fish
	Organ Meat (liver, kidney, heart, blood-based food.)
	Flesh meat (beef, goat, pork, rabbit, chicken)
6. Eggs	Chicken eggs
7. Dark green leafy vegetables	cassava leaves, amaranth leaves, spinach
8. Other vitamin A-rich fruits and vegetables	Pumpkin, tree tomatoes, passion fruits, ripe mango, ripe papaya, carrots, orange sweet potatoes
9. Other vegetables	cabbages, eggplant, cauliflower, tomatoes
10. Other fruits	apple, pineapple, watermelon, avocado, banana, orange, lemon, guava
