# Supplementary material for: Dietary diversity and associated factors among pregnant women in the Southern Province of Rwanda: A facility-based cross-sectional study
Source: PLoS One. 2024 Feb 23;19(2):e0297112. doi: 10.1371/journal.pone.0297112 (PMC10889653; doi:10.1371/journal.pone.0297112)
Supplement: S2 Table — (PDF) [file pone.0297112.s002.pdf]

Questions used to assess nutritional knowledge of pregnant women

Question	Score	Correct answer
1. How should a pregnant woman eat compared to a non-pregnant woman?<br>*Eat more frequently (eat more times each day)*<br>*Eat more food (at each meal)*<br>*Eat more protein-rich foods*<br>*Eat more iron-rich foods*<br>*Use iodized salt when preparing meals*<br>*Eat more fibre-rich foods*<br>*Eat more calcium-rich foods*	1 = Yes<br>0= No<br>0= Don't know	Yes, for each statement
2. Most women would benefit from two types of supplements, or tablets, during pregnancy. Which are they?<br>*Iron supplements*<br>*Folic acid supplements*	1 = Yes 0= No<br>0= Don't know	Yes, for each statement
3. What is the health benefit of taking folic acid supplements/tablets?<br>*a. For normal development of the nervous system of the unborn baby (brain)*<br>*b. For normal development of the nervous system of the unborn baby (spine)*<br>*c. To prevent birth defects/abnormalities in the nervous system of the unborn baby (brain), spine and skull)*<br>*d. To prevent birth defects/abnormalities in the nervous system of the unborn baby (spine)*<br>*e. To prevent maternal anaemia*	1 = Yes 0= No<br>0= Don't know	Yes, for each statement
4. Have you heard about iron deficiency anaemia?	1 = Yes 0= No if no, skip to Q 45	Not scored

5. If Yes: Can you tell me how you can recognize someone who has anaemia?<br>1. Less energy/weakness<br>2. Paleness/pallor<br>3. Spoon nails/bent nails (koilonychias)<br>4. More likely to become sick<br>5. Losing weight<br>6. Having dizziness	1 = Yes<br>0= No<br>99= Don't know	Yes, for each statement
6. How can anaemia be prevented?<br>1. Eat iron-rich foods/have a diet rich in iron<br>2. Eat Vitamin-C-rich foods during or right after meals<br>3. Take iron supplements if prescribed<br>4. Treat other causes of anaemia – seek healthcare assistance.	1 = Yes<br>0= No<br>99= Don't know	Yes, for each statement
7. How many food groups should pregnant women eat every day?	Five or more =1<br>Less than five =0<br>Don't know =0	Five or more
8. How many main meals should a pregnant woman take?	Three or more= 1<br>Less than 3= 0<br>Don't know= 0	Three or more
9. How many main snacks should a pregnant woman take?	Two or more= 1<br>Less than 2= 0<br>Don't know= 0	Two or more
10. It is beneficial to consume snacks to avoid starving for a long time during pregnancy?	1 = Yes 0= No<br>Don't know= 0	yes
11. Appropriate weight gain during pregnancy indicates adequate and balanced nutrition	1 = Yes 0= No<br>Don't know =0	Yes
12. Nutrients deficiency during pregnancy could affect the health status of mothers and baby	1 = Yes 0= No<br>Don't know =0	Yes

13.	Underweight mother can affect foetal well-being and growth	1 = Yes 0= No<br>99= Don't know	Yes
14.	If a woman was a normal weight before pregnancy, how much weight she should gain during pregnancy?	Less than 11.5=0<br>More than 16=0<br>Between 11.5 kg and 16.0 kg =1<br>Don't know =0	Between 11.5 kg and 16.0 kg
