# Supplementary material for: Dietary diversity and associated factors among pregnant women in the Southern Province of Rwanda: A facility-based cross-sectional study
Source: PLoS One. 2024 Feb 23;19(2):e0297112. doi: 10.1371/journal.pone.0297112 (PMC10889653; doi:10.1371/journal.pone.0297112)
Supplement: S3 Table — (PDF) [file pone.0297112.s003.pdf]

Dietary Diversity score of pregnant women in the Southern Province

**Dietary diversity score (**N = 606)			
**Dietary diversity score**	**Frequency**	**%**	**Cumulative %**
1	29	4.8	4.8
2	86	14.2	19.0
3	115	19.0	38
4	109	18.0	56.0
5	88	14.5	70.5
6	78	12.9	83.3
7	46	7.6	90.9
8	24	4.0	94.9
9	21	3.5	98.4
10	10	1.6	100
